# Microbes increase thermal sensitivity in the mosquito *Aedes aegypti*, with the potential to change disease distributions

**DOI:** 10.1371/journal.pntd.0009548

**Published:** 2021-07-22

**Authors:** Fhallon Ware-Gilmore, Carla M. Sgrò, Zhiyong Xi, Heverton L. C. Dutra, Matthew J. Jones, Katriona Shea, Matthew D. Hall, Matthew B. Thomas, Elizabeth A. McGraw

**Affiliations:** 1 Department of Entomology & The Center for Infectious Disease Dynamics, The Pennsylvania State University, University Park, Pennsylvania, United States of America; 2 School of Biological Sciences, Monash University, Melbourne, Victoria, Australia; 3 Department of Microbiology and Molecular Genetics, Michigan State University, East Lansing, Michigan, United States of America; 4 Department of Biology & The Center for Infectious Disease Dynamics, The Pennsylvania State University, University Park, Pennsylvania, United States of America; University of Cincinnati, UNITED STATES

## Abstract

The mosquito *Aedes aegypti* is the primary vector of many disease-causing viruses, including dengue (DENV), Zika, chikungunya, and yellow fever. As consequences of climate change, we expect an increase in both global mean temperatures and extreme climatic events. When temperatures fluctuate, mosquito vectors will be increasingly exposed to temperatures beyond their upper thermal limits. Here, we examine how DENV infection alters *Ae*. *aegypti* thermotolerance by using a high-throughput physiological ‘knockdown’ assay modeled on studies in *Drosophila*. Such laboratory measures of thermal tolerance have previously been shown to accurately predict an insect’s distribution in the field. We show that DENV infection increases thermal sensitivity, an effect that may ultimately limit the geographic range of the virus. We also show that the endosymbiotic bacterium *Wolbachia pipientis*, which is currently being released globally as a biological control agent, has a similar impact on thermal sensitivity in *Ae*. *aegypti*. Surprisingly, in the coinfected state, *Wolbachia* did not provide protection against DENV-associated effects on thermal tolerance, nor were the effects of the two infections additive. The latter suggests that the microbes may act by similar means, potentially through activation of shared immune pathways or energetic tradeoffs. Models predicting future ranges of both virus transmission and *Wolbachia’s* efficacy following field release may wish to consider the effects these microbes have on host survival.

## Author summary

Changes in global climate, which include higher temperatures and more frequent extreme temperature events, are expected to cause dramatic shifts in the distributions of infectious diseases. The geographic range of the mosquito *Aedes aegypti* continues to expand, risking greater incidence of viral diseases including dengue (DENV), Zika, chikungunya, and yellow fever. One emerging solution to control these viruses is the release of the insect bacterium *Wolbachia*, whose infection in mosquitoes reduces virus transmission to humans. However, the effects of rising temperatures on the efficacy of this tool are unclear. Here, we studied whether DENV and *Wolbachia* can alter the thermal sensitivity of the mosquito *Ae*. *aegypti* by using a heat-based physiological assay. We demonstrate that, separately, DENV and *Wolbachia* infections increase mosquito thermal sensitivity, causing more rapid death when mosquitoes are exposed to extreme heat. The impacts of the microbes on mosquito thermal sensitivity were similar but not additive, suggesting they effect the mosquito in similar ways. Our work demonstrates that future global projections of DENV transmission risk and of *Wolbachia’s* potential efficacy may need to consider the impact of these microbes on vector survival.

## Introduction

Mosquitoes are responsible for transmitting a diverse array of human disease-causing viruses such as Zika (ZIKV), chikungunya (CHIKV), West Nile, yellow fever, and dengue (DENV) [1, 2]. The most prevalent of these viruses is DENV. Transmitted by the mosquito *Aedes aegypti*, DENV is responsible for an estimated 390 million cases of dengue fever globally each year [3, 4]. While usually associated with a self-limiting febrile illness, DENV can also cause severe disease that may result in death [4–6]. Without effective antiviral drugs or a vaccine for DENV, ZIKV, or CHIKV, vector control has remained the primary strategy for reducing the incidence of these human diseases [7, 8]. Traditionally, such strategies have relied on insecticide use and larval habitat reduction. A more recent and promising approach involves the use of the insect endosymbiotic bacterium *Wolbachia pipientis* [9]. This self-spreading, vertically inherited bacterium has been transinfected into *Ae*. *aegypti*, where it is being released globally into wild populations for biological control (biocontrol) because *Wolbachia* limits the replication of viruses inside the mosquito, including DENV [10, 11].

*Ae*. *aegypti* is a highly anthropophilic species, restricted to regions with human settlements, where it breeds in human-made containers inside and near housing. The increasing incidence of dengue fever globally is in part due to the ever-expanding geographic range of the vector [5, 12]. Aided by increasing urbanization and climate change, 50% of the world’s population is expected to live in association with *Ae*. *aegypti* by 2050 [13]. Changes in global temperature will shift the map of dengue fever in the following two ways: by increasing risk in previously temperate areas and reducing risk in some regions that exceed mosquito thermal optima [14–16]. The operative temperature range for *Ae*. *aegypti* is between 15.0 and 35.0°C [15], and temperatures outside this range can cause reductions in survival and reproduction and can impact developmental time between stages (i.e., eggs, larva, pupae) [17]. Because the mosquito body temperature is entirely dependent on their environment, they are highly susceptible to various aspects of thermal stress [16]. Thermal stress can be triggered in response to rising average temperatures, as well as extreme climatic events such as heat spikes [18] that are expected to result from greater climate variability.

The individual thermal optima of DENV and *Wolbachia* will also affect global distributions of disease. Warmer ambient temperatures have been associated with increased viral replication in mosquitoes and, consequently, a shorter extrinsic incubation period [19–21]. This parameter represents the time window between when a mosquito first takes an infectious blood meal and when it can transmit virus to a human via a subsequent bite, with a shorter extrinsic incubation period leading to greater transmission rates [22]. Laboratory studies that have reared mosquitoes under different diurnal temperature ranges, which more closely match natural conditions, have also demonstrated changes in *Ae*. *aegypti* susceptibility to DENV [23]. At the molecular level, higher temperatures in mosquito cell culture systems appear to increase viral attachment and entry into cells due to assistance from heat shock proteins [24–26]. All of these parameters affect viral population dynamics in the vector that in turn will affect virus transmission rates at the population level [27–29]. In *Drosophila*, higher ambient temperatures have been shown to increase *Wolbachia* replication rates too but can also become lethal depending on the strain [30–35]. *Ae*. *aegypti* infected with the *w*Mel strain of *Wolbachia* exhibit reduced maternal transmission rates in response to heat spikes that can lead to the production of uninfected offspring [36]. More recently, several studies in *Drosophila* species have demonstrated that *Wolbachia* infection can change the host insect’s thermal preference [37], of which the directionality varies by bacterial strain [38]

More broadly, infection in invertebrates has been shown to substantially increase host or vector susceptibility to thermal stress [39–42]. A recent study conducted by Hector et al., 2019 showed that *Daphnia magna* (water flea) infected with the bacterial pathogen *Pasteuria ramosa* exhibit a reduction in thermal limits up to 2°C. In *Drosophila melanogaster*, immune activation induced by bacterial challenge was shown to affect the temperature at which physiological failure occurred, reducing the overall thermal tolerance (i.e. critical thermal maximum) of the host [39]. Parallel studies have not been carried out for the major mosquito vectors of human disease-causing viruses. In the case of *Wolbachia*, a single study in *Ae*. *aegypti* has revealed that exposure to heat stress made the vector susceptible to starvation in the presence of the symbiont [43]. Both DENV and *Wolbachia* are pervasive throughout mosquito tissues [44, 45] and, therefore, have substantial potential to affect host physiology either directly at the cellular level or indirectly through physiological tradeoffs resulting from activation of the vector’s immune response [46, 47].

Laboratory-based physiological performance assays are commonly used in invertebrates to characterize thermal tolerance [39, 48, 49]. By use of either dynamic or static regimes, these assays provide physiological parameter estimates to inform species distribution models (SDMs). SDMs are important tools used for predicting changes in climate and the response of species and habitats to environmental perturbations. One of the climatic factors commonly used in SDMs is temperature, as it is a major determining factor in the fitness of ectotherms like insects. Critical thermal maxima experiments have been shown to effectively predict an organism’s geographic range [50–54]. More specifically, studies using these experimental assays have suggested that tolerance to extreme heat events near upper critical limits is more indicative of species distributions than tolerance to average daily temperatures [50]. In this study, we used a thermal knockdown assay at a temperature near mosquito upper thermal limits to examine the impact of DENV and *Wolbachia* infection, singly and in coinfection, on *Ae*. *aegypti* thermal tolerance. We hypothesized that the two agents would increase mosquito sensitivity to heat. Any such effect would have the potential to mediate global disease distributions as well as the geographic range over which *Wolbachia*-based biocontrol may be effective.

## Results

### DENV-infected mosquitoes have greater theromosenstivity

**Fig 1:**
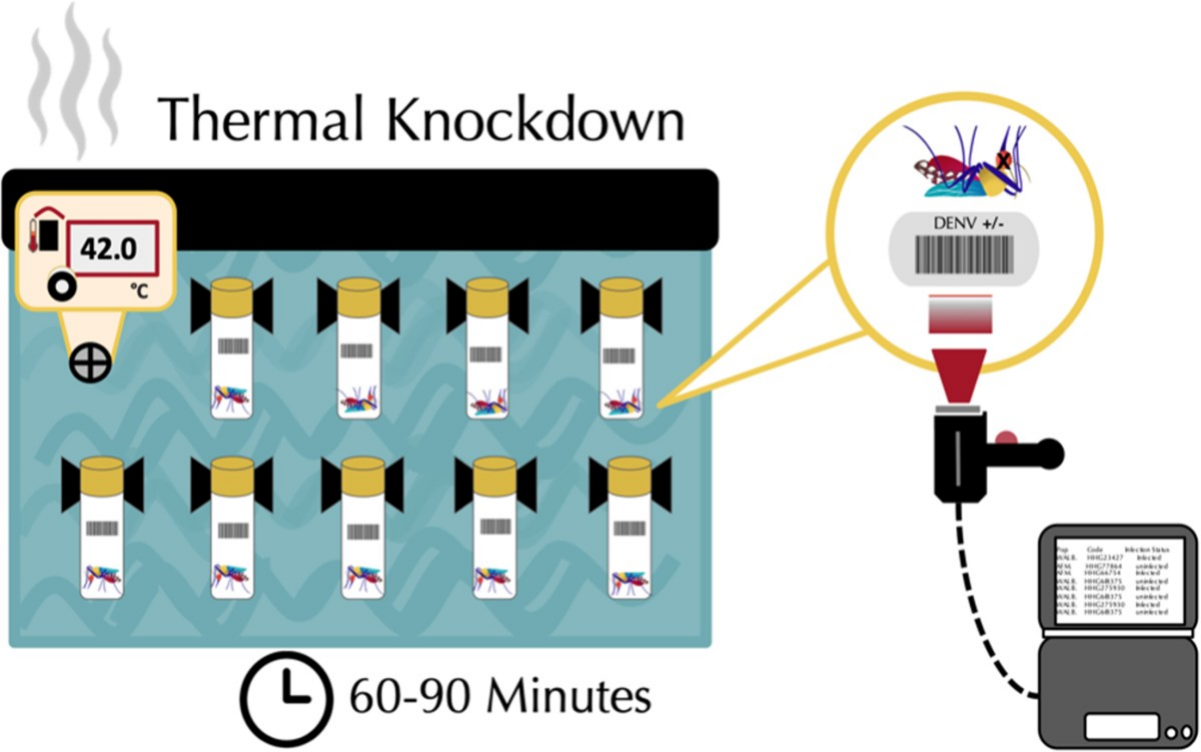
Experimental setup to measure thermal sensitivity of DENV- and *Wolbachia-*infected mosquitoes.

By immersing individual mosquitoes in glass vials in water heated to 42°C, which is beyond the critical maximum temperature, and measuring their time to immobilization, we obtained a measure of ‘knockdown’ (KD) time (Fig 1). This assay and others like it are commonly used in *Drosophila* species to obtain estimates of thermal sensitivity but has not previously used to study mosquitoes.

To test whether DENV and or *Wolbachia* infection alter mosquito thermal sensitivity, we submerged mosquitoes (+/-*Wolbachia*, +/-DENV) in glass vials in a water bath heated to 42°C, representing the upper critical thermal limit (CT_max_) for the mosquitoes, as determined by pilot assays. We then visually monitored the time it took for mosquitoes to become immobilized (on their backs), and this ‘knockdown’ (KD) time was recorded using a barcode scanner.

**Fig 2:**
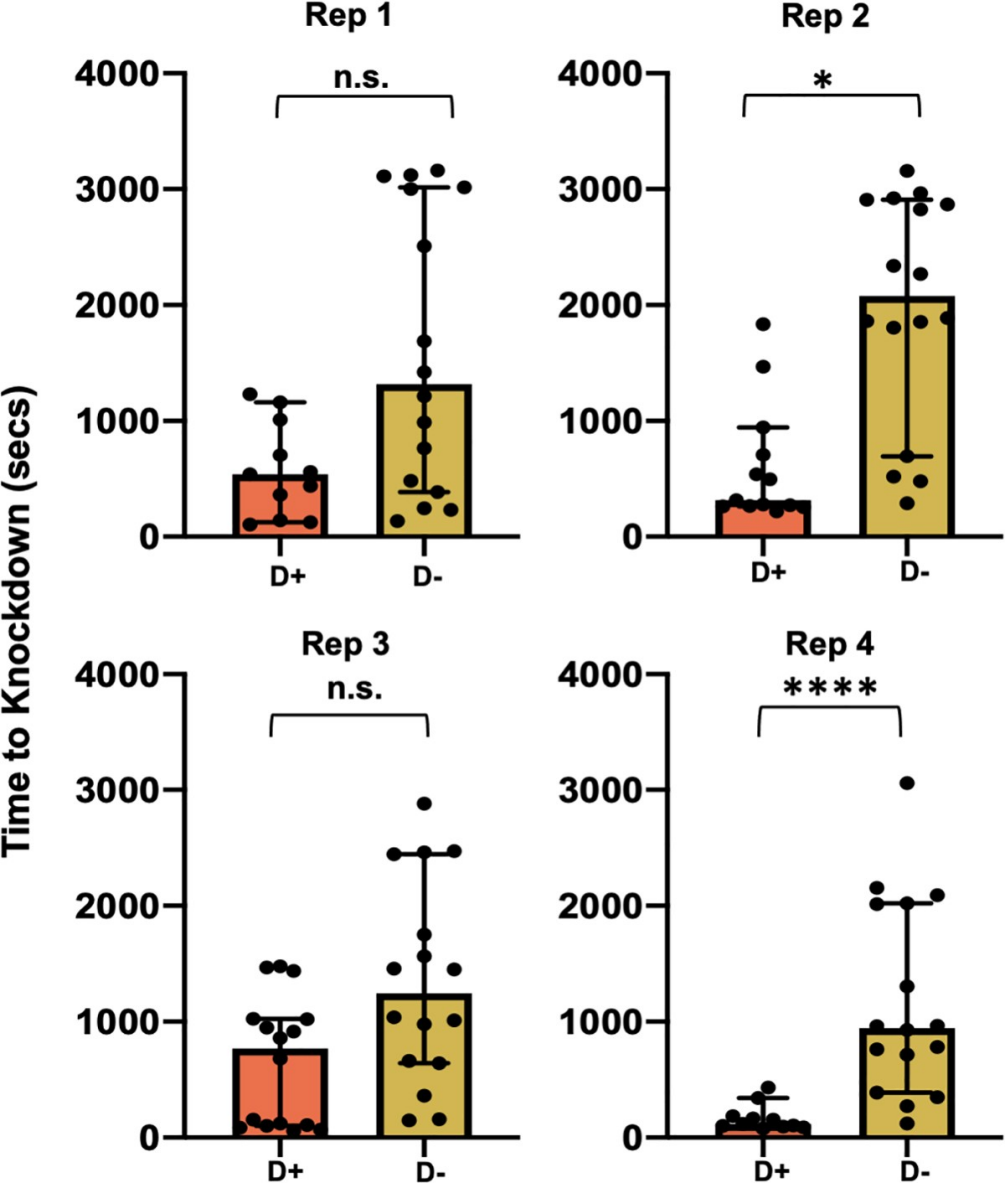
Thermal limits under viral infection. Knockdown time is expressed in seconds for DENV-infected (D+) and DENV-uninfected (D-) mosquitoes with no *Wolbachia* present in either treatment. Each replicate (4) contained 20 individuals per treatment (40 total per block). Box plots represent individual replicate medians and confidence intervals. Both the factors ‘DENV Infection’ (*p*<0.001) and ‘Replicate’ (*p* = 0.035) were significant by ANOVA. *p*-values report Tukey’s post hoc comparison for each replicate.

First, we fed DENV to 9-day-old mated female mosquitoes via a blood meal and then allowed the virus to replicate for 8 days before performing the knockdown assay. Age-matched controls were fed virus-free blood of the same donor and stock. Mosquitoes infected with DENV showed a greater sensitivity to heat (Fig 2) (according to a generalized linear model; S1 Table: ‘DENV Infection’: F = 22.46, df = 2, *p*<0.0001), an effect observed to vary among temporal replicates (“Replicate”: F = 5.07, df = 3, *p* = 0.035). On average, DENV-infected mosquitoes had a median KD time that was 2.9-fold faster than that of DENV-uninfected mosquitoes. Tukey’s post-hoc comparisons indicated that the impact of DENV was significant (*p*<0.0001) for 2 of the 4 temporal replicates (S2 Table). As genetic and environmental effects should be uniform across replicates, differences in the impact of DENV could be explained by day/circadian rhythms. There was, however, no clear trend of KD time decreasing or increasing with time of day/sequential replicate.

### Viral load does not determine time to thermal knockdown

**Fig 3:**
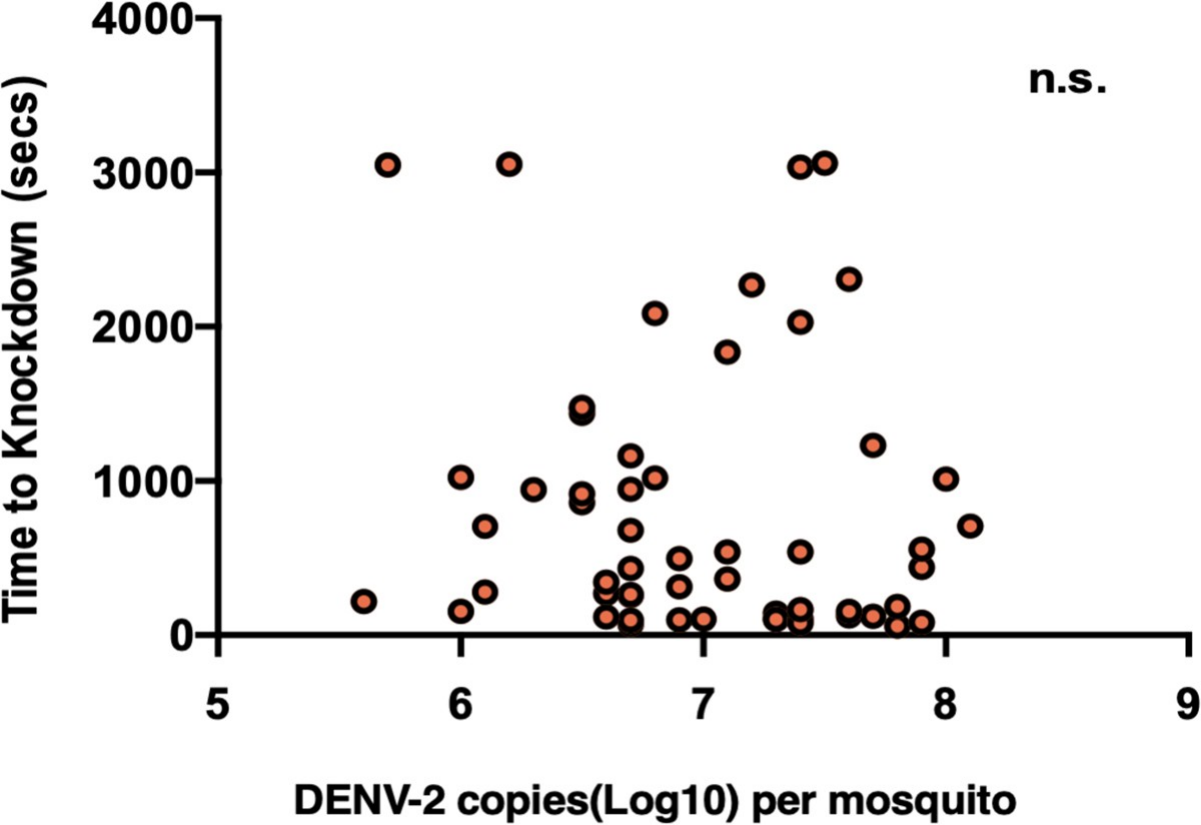
Relationship between viral load and thermal knockdown in mosquitoes. Relationship between knockdown time (seconds) and DENV load (per mosquito) in wildtype (D+/*W*−) mosquitoes. Each point represents a single mosquito with individuals from all 4 replicate experiments. There was no significant relationship (Pearson correlation, *p*>0.05) between knockdown time and DENV load for the pooled set or for individual replicate experiments.

We then examined whether there was a relationship between viral load and time to knockdown (Fig 3), as pathogen load often predicts virulence in many systems, including DENV [55]. Surprisingly, we saw no such relationship between dengue load and knockdown time in our) WT+(Wildtype) line (Pearson correlation, df = 57, *p*>0.05) despite that mosquito body loads ranged from 10^6^ to 10^8^ viral genome copies/mosquito.

### Thermal limits under viral and bacterial infection

**Fig 4:**
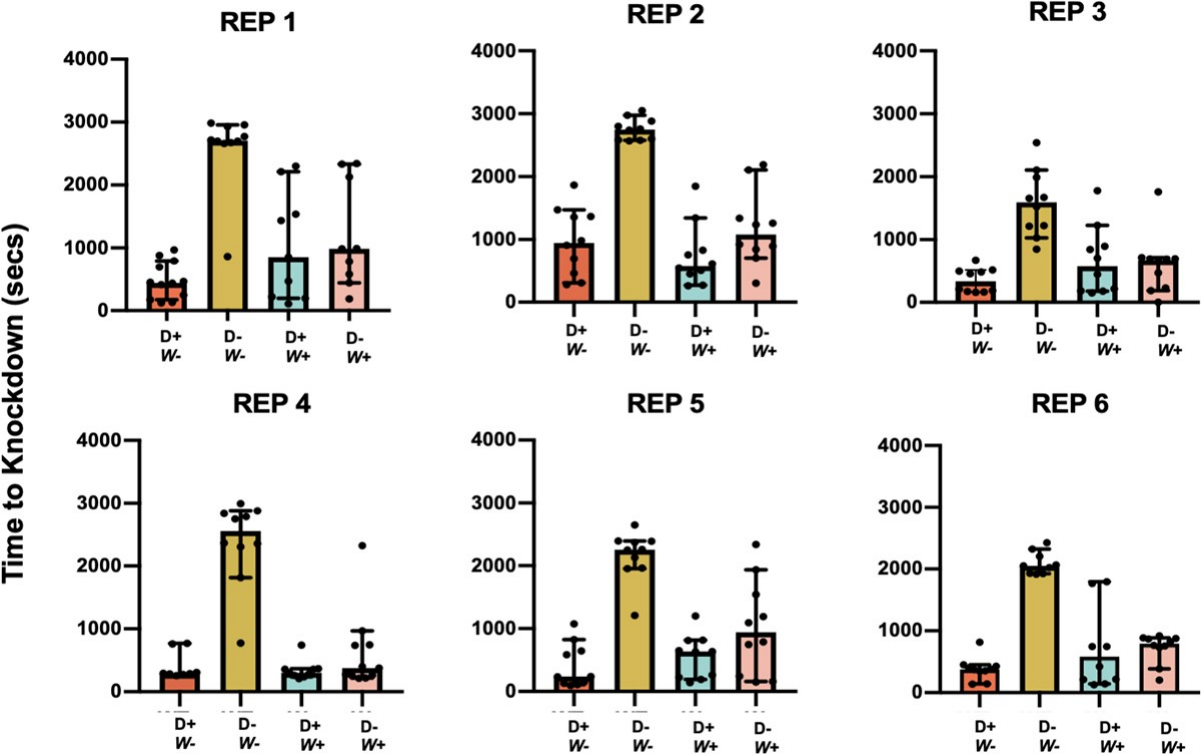
Impact of dual microbe infection on mosquito thermal sensitivity. The effect of *Wolbachia* and DENV infection on knockdown time (seconds) across 6 temporal replicates, each containing 10 mosquitoes per the 4 treatment combinations. Box plots represent individual replicate medians and confidence intervals for wildtype infected (D+/W-) and uninfected (D-/W-) mosquitoes, along with *Wolbachia* infected (*W*+) and uninfected (*W*-*)* individuals. Key Tukey’s *post hoc* comparisons for each replicate are described in the text and S5 Table. In brief, the effects of DENV and *Wolbachia* individually were significant in all 6 replicates, as was the comparison between the single infection (DENV) and the double infection (DENV and *Wolbachia*).

Several *Wolbachia* strains are being released globally [56, 57]. Here, we used *w*AlbB, a strain transferred from *Aedes albopictus* [10] into *Ae*. *aegypti* that has shown promise with respect to reducing incidence of dengue fever following field releases in Malaysia [57]. We assessed the effect of *Wolbachia* infection (*w*AlbB) vs. wildtype on mosquito knockdown time in association with DENV infection (+/-) (Fig 4). In our generalized linear model (DENV + *Wolbachia* + DENV:*Wolbachia* + Rep), ‘DENV infection’ (F = 94.64, df = 1, *p* < .0001), ‘*Wolbachia* infection’ (F = 23.75, df = 1, *p* < .0001), and ‘Temporal Replicate’ (F = 5.05 df = 5, *p* = 0.0002) were all significant, with both infections increasing thermal sensitivity or decreasing knockdown(KD) time (S3 Table). There was also a significant interaction between ‘DENV infection’ and ‘*Wolbachia* infection’ (F = 55.68, df = 1, *p* < .0001). Because ‘Temporal Replicate’ was significant, we then followed with individual ANOVAs for each replicate (S4 Table) so that we could carry out individual Tukey’s post hoc comparisons (S5 Table). In 6/6 replicates, DENV infection significantly reduced KD time. On average, across replicates, the median KD time of DENV-infected mosquitoes (D+*W*-) was 4.5-fold more rapid (Fig 4) than that of uninfected controls (D-*W*-). This knockdown (KD) appears to be faster than that seen in experiment 1 (Fig 2), but comparisons across experiments are not valid given different virus preparations and use of separate mosquito cohorts. *Wolbachia* infection also reduced median KD time in 4/6 replicates (#’s 2, 4–6), conferring a 2.5-fold faster average KD time than WT mosquitoes in the absence of DENV (D-*W*+ vs D-*W*-). Not surprisingly, the double-negative state (D-W*-*) has a longer KD time than D+W+ for all 6 replicates. If *Wolbachia-*mediated blocking was protecting the mosquito from DENV-induced thermal sensitivity, it would be seen in the D+W- vs D+W+ comparison. None of these comparisons were significant for any of the replicates. Additionally, if the two infections were additive, one would expect D+W+ to have a faster KD time than either D+W- or D-W+. None of these comparisons are significant for any of the replicates. However, in only one replicate (#5), the DENV effect is greater than the *Wolbachia* effect (D+W- vs D-W+), increasing KD time. This pattern of DENV potentially being stronger than *Wolbachia* is also in keeping with that *Wolbachia* was significant in 5/6 ANOVAs compared to 6/6 for DENV (S4 Table).

### Microbial load does not determine time to thermal knockdown

**Fig 5:**
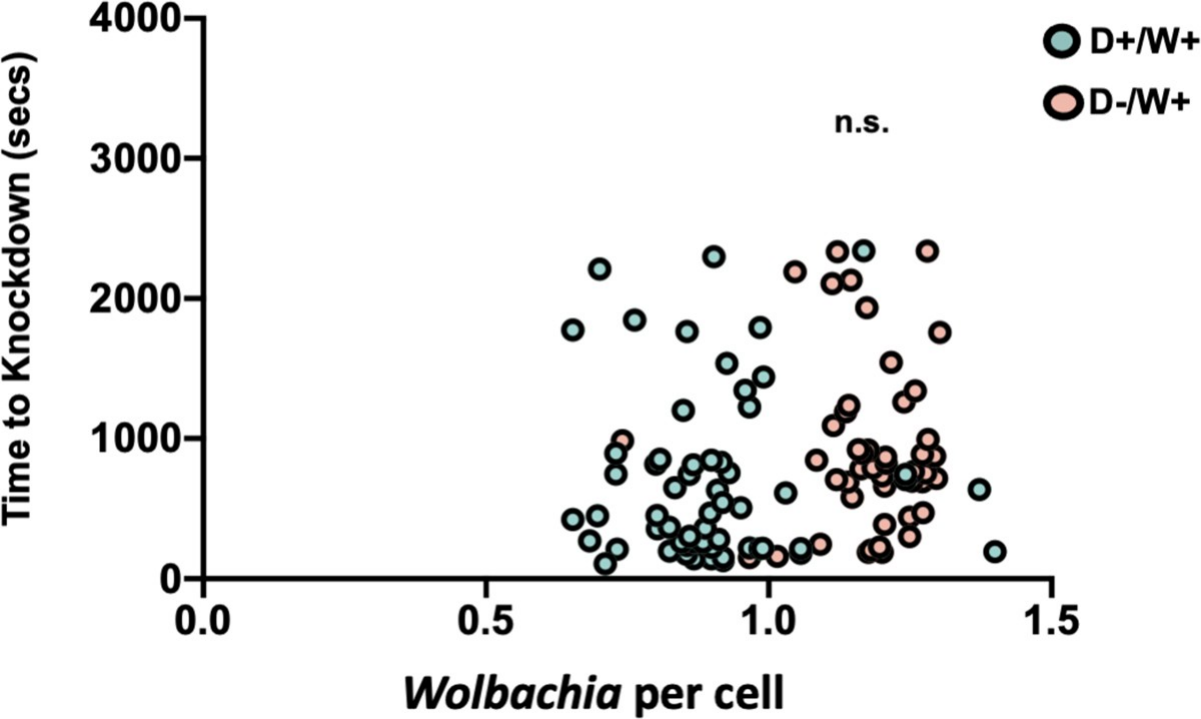
Relationship between bacterial load and time to knockdown in mosquitoes. Each point represents a single mosquito, with individuals from all 4 replicate experiments presented on one graph. There was no significant relationship (Pearson correlation, *p*<0.05) between knockdown time and *Wolbachia* load for the pooled set or for individual replicate experiments analyzed separately (S6 Table).

As in Experiment 1 above, there was no evidence that KD time was determined by DENV load in Experiment 2 (Pearson correlation, df = 98, *p* = 0.2048) (S1 Fig). As for DENV above, we examined whether a relationship existed between total body load of *Wolbachia* and knockdown(KD) time (Fig 5). There was no significant relationship for the data when pooled across replicate (Pearson correlation, df = 117, *p*>0.05) or when analyzed individually (S6 Table). Interestingly, we noted that *Wolbachia* loads were lower when DENV was present (Figs 5 and S2; F = 1.924, df = 116, *p*<0.0001), which may relate to a virus-induced immune killing of *Wolbachia* or resource competition between *Wolbachia* and DENV, as both microbes share similar host resources [58]. We therefore also split the mosquitoes into DENV+ or -, pooled across replicates, and retested for a correlation between *Wolbachia* load and knockdown(KD) time but saw none for either D+*W*+ (*p*>0.05, df = 57) or D-*W*+ (*p*>0.05, df = 46).

## Discussion

The impact of DENV infection on vector thermal sensitivity has implications for global dengue risk under a changing climate. Numerous studies have mapped the likely range of *Ae*. *aegypti* into the future, based on its current occupation of global thermal zones and mechanistic effects of temperature on mosquito and pathogen traits [14, 15]. At lower temperatures, the virus may fail to replicate fast enough to traverse the mosquito body and be transmitted [59], reducing transmission risk in some areas. At slightly higher temperatures, the virus may replicate faster until reaching a performance maximum of its own [23]. Our work suggests an additional factor may affect viral success in a temperature-dependent manner—the impact on mosquito survival.

Although the assay used here represents a simplified model of heat stress, future climate models point to increasing frequencies of extreme temperature events, making short exposures to high temperatures a threat to the survival of DENV and Wolbachia infected mosquitoes. Substantial evidence from *Drosophila* and other insects suggests that their ability to survive heat stress is highly predictive of an insect’s current distribution and therefore also likely its future range [48, 49]. Thermal sensitivity (KD) measures, specifically, have been shown to be a relevant proxy for fitness under field relevant conditions [50]. In agreement with this, variation seen in *Drosophila* populations for measures such as thermal knockdown (KD) time is predictive of an ability to respond to artificial selection for thermal resistance/sensitivity, and as a result single trait physiological measures like these can be used effectively for developing SDMs [51, 60].

The findings for *Wolbachia w*AlbB indicate that the symbiont increases mosquito thermal sensitivity, although not to the same level as DENV. Both agents infect a diversity of tissues throughout the body [11] and, although not cytotoxic, evoke an immune stress response in *Ae*. *aegypti* [61, 62]. Most studies suggest that DENV has little effect on host fitness except in rare cases [63] Additionally, because *Wolbachia* lacks a complete set of metabolic pathways, it represents an energetic drain on host resources, including amino acids [64]. Three outcomes were possible for the relationship between DENV and *Wolbachia* coinfection and KD time, namely, protective, additive, or similar. A protective effect might have been expected given that *Wolbachia*-mediated blocking is known to limit DENV loads in the body [61] (also seen in this study S3 Fig), although we did not see a correlation between *Wolbachia* and DENV loads (r = -0.052, *p* = 0.96). This would only be the case if increasing loads of DENV led to faster KD times, but we saw no relationship. An additive effect might have suggested that the two infectious agents acted on independent aspects of mosquito physiology and both cause thermal sensitivity. We saw no difference in thermal sensitivity between the single and doubly infected mosquitoes. We also saw no relationship between *Wolbachia* load and KD time, like DENV, indicating that having more of either agent did not lead to greater virulence. Taken together, our data agree with a model of the symbiont and virus acting via a shared mechanism. One possible explanation is that the microbes activate similar innate immune pathways [47, 61, 65]. The effect of this activation may have direct pleiotropic effects on thermal tolerance or act through energetic tradeoffs. Interestingly, any triggering of the heat shock response by DENV infection [62] itself was not powerful enough, or long lasting enough, to mitigate the impact of either DENV or *Wolbachia* on KD times.

To capture the impact of viral infection upon mosquito thermal limits, we assayed individuals in the thermal KD setup by using a static tolerance assay as compared to a dynamic assay, in which the insect is gradually exposed to ramping temperatures until thermal knockdown (KD) is achieved. However, whether these two physiological assays provide comparable measures for heat tolerance has been questioned [66]. The overall outcome for both assays is dependent on the duration of heat exposure and the temperature at which thermal stress occurs [50]. In nature, ambient temperatures are rarely constant, and mosquitoes and their pathogen are subjected to temperatures that may fluctuate throughout the day [67]. Dynamic assays have been promoted for their ecological relevance due to their gradual increase in temperature versus an acute exposure to high temperature [68, 69]. A study conducted by Rezende et al., 2014 looked at this interaction between the intensity of heat stress and exposure duration through the development of thermal tolerance landscapes (TTLs), which depict the parametrization of survival time as a function of constant temperatures plus thermal exposure duration. In a past study, this group showed that TTLs are able to predict survival in thermally variable environment’s when using empirical data that have incorporated either dynamic or static measures of thermal stress.

To mediate any discrepancies between dynamic and static assays, Jørgensen et al., 2019 developed a model that allowed for the direct comparison of static and dynamic measures of heat tolerance from KD data obtained from *Drosophila* [50]. They showed that a dynamic CTmax, at any given heating rate, can be effectively modeled from static measurements like ours and used to predict species distributions [50]. Although the assay we used is a simplified representation of heat stress, future climate models point to increasing frequencies of extreme temperature events, making short exposures to high temperatures relevant. Future studies should examine these effects in the context of diurnal temperature range variability reflecting natural temperatures and repeated exposure to heat shock events.

Transient exposure exceeding the mean temperature beyond thermal optima can have costly fitness effects on an individual [70, 71]. Maximum thermal tolerance in an individual is dependent on cell performance and actions of different systems, including respiratory, circulatory, and nervous [72, 73]. High heat denatures enzymes and disturbs cellular membranes that thus impact cellar processes that the insect may rely on to function [72, 73]. Our measurement of thermal limits may have some confounding impacts on desiccation or starvation stress [74]. However, studies with *Drosophila* show that an exposure time of 60–90 minutes was not sufficient to trigger either a starvation or desiccation response [69]. Additionally, in our system, these responses were likely minimized because mosquitoes were provided sugar water and vials were not sealed until 25 minutes before their thermal knockdown with humid air [69]. Future research examining the interaction of multiple related stressors will provide interesting insight into the range of responses that might be seen in wild populations with more complex environments under predicted climate shifts. Additionally, our study did not allow for the host to respond behaviorally to heat stress. In the field, mosquitoes may use various responses, such seeking shade or cooler areas, when thermally stressed [75]. In general, they may also close spiracles to reduce dehydration and activate a series of pathways at the cellular level, including heat shock [62, 76].

Daily and seasonal fluctuation is higher in temperate regions, whereas tropical areas experience less seasonality [77]. Species from temperate areas and high-altitude regions have broader thermal tolerance thresholds, as they can tolerate warming due to their ability to respond to variable temperatures [78]. Tropical species like mosquitoes, however, are living close to their optimal temperatures for performance [67, 79] and experience little variation in daily and seasonal fluctuation [77, 80, 81]. The impact of global climate change on mosquito-borne disease will depend strongly on species thermal history and their overall tolerance and ability to withstand change and adapt [16, 70, 82, 83]. The context of our findings should be considered within the realm of local thermal adaption, which may lead to different responses between populations, and how they respond to thermally taxing conditions. Furthermore, environmental conditions experienced across the larval stage of the mosquito can have an impact on mosquito response to thermal stress [84]. Thermal acclimation can a happen within a population and affect subsequent adult traits that can happen irrespective of any local adaption [67, 79]. In our case, mosquitoes were lab reared at constant temperatures, but if larval rearing conditions varied within a range of thermal conditions or mosquitoes were locally adapted, we may conclude that KD could differ depending on their prior thermal exposure.

With this study, we now add increased thermal sensitivity to a list of heat-associated effects for adult *Wolbachia*-infected mosquitoes that include increased susceptibility to starvation [43], reductions in maternal transmission rates of *Wolbachia*, and loss of cytoplasmic incompatibility [36]. These temperature-associated effects may reduce the competitiveness of *Wolbachia*-infected mosquitoes in extremely hot or variable environments and affect the efficacy of the biocontrol strategy. Additionally, *Wolbachia* transinfected into mosquitoes induces fitness costs that produce bistable frequency equilibria, which limit how the symbiont spreads in populations when established from low infection frequencies [85]. Increased thermal sensitivity may contribute to these net fitness costs and bistable equilibria. Naturally occurring *Wolbachia* infections commonly evoke a lower immune response, potentially from a longer history of coevolution with their hosts [86]. One solution for protecting *Wolbachia’s* use for biocontrol in hotter regions might involve adapting mosquito: *Wolbachia* pairings to higher temperatures or for reduced immune responses in the laboratory by using artificial selection before release into the field.

Several factors may have affected our study design or should be considered in future studies. First, the effect of infection on KD time may rely heavily on the combination of *Wolbachia* strain, virus and mosquito genotypes, and their past histories of thermal adaptation. Additionally, some genotypes of both the host and pathogen may be better able to compensate for thermal stress [40]. Thermal tolerance should therefore be examined for combinations of key circulating viruses and *Wolbachia* release strains in diverse *Ae*. *aegypti* populations from different global regions. Second, factors like mosquito age at exposure, reproductive status, body size or nutritional status, blood meal history, and other coinfecting microbes/the microbiome may also play a role in thermal tolerance. Third, as for the Drosophila literature [48], it will be important to assess how these laboratory measures of thermal sensitivity relate to fitness measures in the field and how the interaction between viral infection and temperature affect the distribution of virus transmission. Fourth, it would be interesting to assess whether thermal sensitivity effects due to *Wolbachia* are also present in the larval stage. If so, they may affect the successful field release of *Wolbachia* via egg stage [87].

In conclusion, our work suggests that DENV- and *Wolbachia*-induced increases in mosquito thermal sensitivity may limit the geographic range of the virus’s transmission to humans and the ability of the symbiont to be used for biocontrol. We suggest that future models predicting dengue distribution may also need to incorporate the interaction between virus and vector survival to be accurate, particularly at the edge of a mosquito’s distribution where the potential impact of these microbes would likely be greatest in hotter and more thermally variable regions of the mosquitos’ range.

## Materials & methods

### Mosquitoes

Within a year of this study, the *w*AlbB *Wolbachia*-infected *Ae*. *aegypti* line was backcrossed for 7 generations to a wild-caught mosquito line (AFM-Wildtype [WT]), that was collected from the field in Mérida, Mexico, by Pablo Manrique. This process homogenized the nuclear genetic background with the field line but would have retained the mitochondria from the *Wolbachia*-infected line. We used this continuously maintained line (~1 year) for the DENV+/- experiments. Furthermore, because this population was not naturally infected with *Wolbachia*, we also used it as a negative control for subsequent *Wolbachia* experiments. Both lines were reared under standard conditions: 12hr light/dark, 26°C, 60% relative humidity, *ad libitum* Tetramin fish food at the larval phase, and 10% sucrose as adults.

### Virus

We used DENV serotype 2 for all experiments, as it has been previously shown to form strong infections in the mosquito in the laboratory [11]. Originally isolated from a patient in East Timor, the ET-300 strain (GenBank EF440433.1), with approximately 20 passages, was used in the assay, as done previously in a study of *Wolbachia*:mosquito:DENV interactions [88]. *Ae*. *albopictus* C6/36 cells were grown at 26°C in RPMI 1640 medium (Invitrogen, Carlsbad, CA) supplemented with 10% fetal bovine serum (FBS), 1× Glutamax (Invitrogen), and HEPES buffer. Cells were first allowed to form monolayers of around 60–80% confluence in T-175 flasks (Sigma Aldrich, St. Louis, MO) and then were inoculated with DENV and maintained in RPMI medium supplemented with 2% FBS. At day 7 post-inoculation, live virus was harvested, titrated via absolute quantification RT-qPCR, and adjusted to a final viral load of 10^7^ DENV copies per ml.

### Mosquito infections with DENV

Before an infectious blood meal, mated, 9-day-old female mosquitoes were sorted into groups of 100 in 68-oz paper cartons. Sucrose was removed from the mosquitoes 24 hrs before oral infection and replaced with water. Double-chamber glass feeders were covered with pig intestine previously immersed in a 10% sucrose solution. Water heated to 37°C was circulated in the outer chamber of the feeders, and a 1:1 mix of defibrinated human blood and the previously titrated DENV virus was placed inside the feeder. The final feed concentration was 2.5e^7^ DENV copies/ml. In parallel, all DENV− mosquitoes were fed a solution containing a 1:1 ratio of blood without virus and RPMI 1640 cell culture media to serve as mock controls. After 24 hrs, all blood fed mosquitoes were identified by visual inspection under chilling and returned to the cartons.

### Thermal knockdown assay

The thermal sensitivity of infected and uninfected mosquitoes was measured using a static heat shock assay (Fig 1) based on previous work in *Drosophila* [89]. KD experiments were carried out at 42°C, as pilot studies indicated this temperature represented the critical thermal maximum of the mosquitoes (S7 Table). Exposure to 42°C led to death in 97% of individuals trialed in the WT line even without the presence of DENV. From the literature, this temperature also represents some of the upper thermal extremes insects may encounter in nature due to global climate change [17]. Mosquitoes were moved 24 hrs after blood feeding to individual 40-ml glass vials with mesh lids topped with cotton balls soaked in 10% sucrose that were changed daily. All vials were housed in environmental chambers maintained at 26°C and 65% relative humidity. Knockdown (KD) assays were performed 8 days after blood feed, allowing time for the virus to disseminate throughout the body and affect mosquito physiology [19]. Before each assay (<25 minutes), the mesh lids to the vials were replaced with solid plastic. The assays themselves were also carried out in the environmental chambers, so air captured in the vials upon sealing was at 65% relative humidity. The vials containing the mosquitoes were then attached to a plastic board in groups of 40 via anchored clips, randomized with respect to treatment. The board with vials was immersed in a water bath heated to 42°C and allowed a 60-second acclimation period. Mosquitoes were then monitored visually for immobility and time to thermal knockdown (KD) was scored using Brady labels and a TriCoder Scanner (Worth Data Inc., Santa Cruz, California). Immobility was confirmed by tapping on the vial while it was still immersed in the water bath. Mosquitoes did not recover after thermal knockdown(KD). The DENV status of all individuals was confirmed by PCR as described below.

### Mosquito nucleic acid extraction

Upon completion of each KD experiment, individual whole mosquitoes were anesthetized by chilling and placed in 1.5-ml microfuge tubes (Sarstedt, Nümbrecht, Germany) containing 300 μl of TRIzol reagent (Invitrogen, Carlsbad, CA, USA) and a 2.8-mm ceramic bead. Samples were homogenized on a Bead Ruptor Elite (Omni International, USA) and then frozen at −80°C. Total RNA was extracted with the Direct-zol RNA 96 Magbead Zymo kit (Zymo Research, Irvine, CA) according to the manufacturer’s protocol. Following this step, the samples were processed using an automatic magnetic bead purification system (MagMAX Express 96 system, Applied Biosystems). RNA was eluted in 50 μl RNase free water. RNA was then treated with 5 units of DNase I (Sigma-Aldrich) at room temperature for 15 min, followed by inactivation with 50 mM EDTA at 70°C for 10 min. To measure *Wolbachia* loads, extractions for both RNA and DNA were performed using the column-based Direct-zol DNA/RNA Miniprep kit. RNA was eluted in 50 μl RNase free water, followed by DNA elution in 50 μl of Direct-zol DNA Elution Buffer. Total RNA and DNA concentrations were determined with a NanoDrop model 2000/2000C (Thermo Scientific, Waltham, MA).

### DENV quantification

DENV virus was quantified using TaqMan Fast Virus 1-step Master Mix (Thermo Fisher Scientific) in 10-μl reaction volumes with DENV-specific primers and probes [90] (S8 Table). The following protocol was used: reverse transcription at 50°C for 5 min, followed by 50 amplification cycles 95°C for 20 sec, and amplification cycling at 95°C for 3 sec and 60°C for 30 sec. A standard reference curve of known quantities of a DENV-2 genomic fragment was used for absolute quantification by qPCR. The DENV-2 genomic fragment was previously inserted into a plasmid and transformed into *Escherichia coli* as described [90]. The linearized and purified fragment was serially diluted ranging from 10^7^to 10^2^ copies and used to create a standard curve of DENV amplification. The standard curve was run in duplicate on each 96-well plate, and the limit of detection was set at 10^2^ copies. All samples were run in duplicate.

### *Wolbachia* quantification

*Wolbachia* load was assessed as previously reported including published primers and probes [90]. In brief, a multiplex qPCR reaction amplifying the target *Wolbachia*-specific surface protein *wsp* and mosquito ribosome subunit 17 housekeeping gene *Rp*S*17* was performed. The *Rp*S*17* gene was used to normalize *wsp* gene copies. Quantitative PCR reactions were run in duplicate and performed in a 10-μl total volume containing 1× Lightcycler 480 Probes Master reaction mix, 5 μM of each *wsp* primers and probe, 2.5 μM each of *Rp*S*17* primers and probe, and 1 μl of DNA template. Cycling was performed using a LightCycler480 Instrument (Roche), with 1 cycle at 95°C for 5 min; followed by 45 amplification cycles of 95°C for 10 s, 60°C for 15 s, and 72°C for 1 s; and a final cooling cycle of 40°C for 10 s. Target to housekeeping gene ratios were calculated using the Livak’s 2^−ΔΔCT^ method relative quantification algorithm in the Lightcycler 480 software (Roche).

### Experimental design and statistical analysis

In the first set of experiments, mosquitoes without *Wolbachia* were infected with DENV (passage 36) as described above and tested for KD in 4 temporal replicates each containing 20 individual mosquitoes per treatment (+/−DENV). All mosquitoes were from the same generation/population of mosquitoes. In the second set of experiments, we explored both *Wolbachia* (+/-) and DENV (+/-) infection. *Wolbachia*-free mosquitoes were drawn from the same population as above, 3 generations hence. As for the first experiment, live virus was cultured to feed to these mosquitoes, from virus passaged 38 times. Any mosquitoes not infected with DENV in DENV+ treatments (~4% in *W*- mosquitoes in experiments 1 and 2 and 53% *W*+ mosquitoes in experiment 2) were excluded from the data analysis. All knockdown data were log transformed before analysis to correct issues with skew. In experiment 2, each of the 4 combinations of infection status was represented by 10 individual mosquitoes in a temporal replicate. These experiments were then replicated 6 times (blocks). All statistics were carried out using JMP Version 4 for Mac (SAS Institute Inc., Cary, NC, USA). Knockdown (KD) time was examined using general linear models with ‘DENV infection,’ ‘temporal replicate,’ and ‘*Wolbachia* infection’ as fixed effects where relevant. The relationships between each DENV load and *Wolbachia* load and KD time were examined with Pearson’s correlation.

## Supporting information

S1 Table Impact of DENV infection alone on KD time. ANOVA for Fig 2, with ‘DENV infection status’ and ‘temporal replicate’ as factors.
(DOCX)

S2 Table Impact of DENV infection alone on KD time for each replicate independently. Tukey’s post hoc comparisons for Fig 2 for DENV infection status (D+/-).
(DOCX)

S3 Table Impact of DENV and *Wolbachia* co-infection on KD time. GLM for KD time for Fig 4 including ‘Rep’, ‘DENV status’, and ‘*Wolbachia* status’ as factors.
(DOCX)

S4 Table Impact of DENV and *Wolbachia* co-infection on KD time for each replicate independently. ANOVAs for knockdown time for Fig 4 for each of the six individual temporal replicates with ‘DENV status’ and ‘Wolbachia status’ as factors.
(DOCX)

S5 Table Impact of DENV and *Wolbachia* infections in mono and co-infection on KD time for each replicate independently. Tukey’s post hoc comparisons of KD time for Fig 4 for all combinations of DENV (D+/-) and *Wolbachia* (W+/-) infection status.
(DOCX)

S6 Table Correlation between *Wolbachia* load and KD time. Summary of individual rep correlations between *Wolbachia* load and KD time for Fig 4.
(DOCX)

S7 Table Survival during pilot knockdowns at varying temperatures (DENV+/-). Summary of t-tests from pilot experiments conducted at varying temperatures showing average time to knockdown for dengue infected (D+*W*-) and uninfected (D-*W*-) individuals. All temperatures resulted in a knockdown phenotype, but 42°C led to death in 97% of individuals trialed in the WT line even without the presence of DENV. 42°C also allowed resulted in faster knockdown times during the assay.
(DOCX)

S8 Table Primers and probes for dengue virus serotype 2 detection. (DOCX)

S1 Fig Relationship between viral load and thermal knockdown in mosquitoes for Fig 4. KD time (seconds) vs. DENV load (per mosquito) in Wildtype (*W*-) mosquitoes. Each point represents a single mosquito. Data include all individuals across 6 replicate experiments. There was no significant relationship (Pearson’s correlation, P = 0.204) between KD time and DENV load.
(DOCX)

S2 Fig Reduction of *Wolbachia* load in the presence of DENV for Fig 4. Average *Wolbachia* loads with (D+*W*+) and without (D-*W*+) DENV. Data are pooled across 6 replicate experiments. Graphs depict mean ± sem *Wolbachia* per host cell. *Wolbachia* load is reduced in the presence of DENV infection (*df* = 116, F-Ratio = 1.92, *p*<0.0001).
(DOCX)

S3 Fig Evidence of *Wolbachia*-mediated blocking of DENV for Fig 4. Average DENV loads for *Wolbachia* infected (D+*W+*) and uninfected (D+W-). Data are pooled across the 6 replicate experiments. Graphs depict mean ± standard error. Loads are reduced in the presence of *Wolbachia* (*df* = 115, F = 13.32, *p*<0.001).
(DOCX)
