# Supplementary material for: Microbes increase thermal sensitivity in the mosquito Aedes aegypti, with the potential to change disease distributions
Source: PLoS Negl Trop Dis. 2021 Jul 22;15(7):e0009548. doi: 10.1371/journal.pntd.0009548 (PMC8297775; doi:10.1371/journal.pntd.0009548)
Supplement: S1 Table — ANOVA for Fig 2, with ‘DENV infection status’ and ‘temporal replicate’ as factors. (DOCX) [file pntd.0009548.s001.docx]

**Supplemental Table 1. Impact of DENV infection alone on KD time.** ANOVA for Fig. 2, with ‘DENV infection status’ and ‘temporal replicate’ as factors.

| **Effect** | **Nparm** | ***df*** | **Sum of**  **Squares** | **F-Ratio** | ***p*-value** |
| --- | --- | --- | --- | --- | --- |
| DENV Status | 2 | 2 | 28842303 | 22.46 | <.0001* |
| Rep | 3 | 3 | 5717907 | 2.96 | 0.035* |
