# Supplementary material for: Microbes increase thermal sensitivity in the mosquito Aedes aegypti, with the potential to change disease distributions
Source: PLoS Negl Trop Dis. 2021 Jul 22;15(7):e0009548. doi: 10.1371/journal.pntd.0009548 (PMC8297775; doi:10.1371/journal.pntd.0009548)
Supplement: S2 Table — Tukey’s post hoc comparisons for Fig 2 for DENV infection status (D+/-). (DOCX) [file pntd.0009548.s002.docx]

**Supplemental Table 2. Impact of DENV infection alone on KD time for each replicate independently.** Tukey’s post hoc comparisons for Fig. 2 for DENV infection status (D+/-).

| **Rep** | **Comparisons** | **Adjusted *p*-value** |
| --- | --- | --- |
| 1 | D+ vs. D- | 0.22 |
| 2 | D+ vs. D- | 0.011 |
| 3 | D+ vs. D- | 0.058 |
| 4 | D+ vs. D- | <0.0001 |
