# Supplementary material for: Microbes increase thermal sensitivity in the mosquito Aedes aegypti, with the potential to change disease distributions
Source: PLoS Negl Trop Dis. 2021 Jul 22;15(7):e0009548. doi: 10.1371/journal.pntd.0009548 (PMC8297775; doi:10.1371/journal.pntd.0009548)
Supplement: S3 Table — GLM for KD time for Fig 4 including ‘Rep’, ‘DENV status’, and ‘Wolbachia status’ as factors. (DOCX) [file pntd.0009548.s003.docx]

**Supplemental Table 3. Impact of DENV and *Wolbachia* co*-*infection on KD time.** GLM for KD time for Fig. 4 including ‘Rep’, ‘DENV status’, and ‘*Wolbachia* status’ as factors.

| **Effect** | **Nparm** | ***df*** | **Sum of**  **Squares** | **F-Ratio** | ***p*-value** |
| --- | --- | --- | --- | --- | --- |
| Rep | 5 | 5 | 2.83 | 5.05 | 0.0002* |
| DENV status | 1 | 1 | 10.61 | 94.64 | <.0001* |
| Rep x DENV status | 5 | 5 | 0.44 | 0.79 | 0.55 |
| *Wolbachia* status | 1 | 1 | 2.66 | 23.75 | <.0001* |
| Rep x *Wolbachia* Status | 5 | 5 | 0.49 | 0.88 | 0.49 |
| DENV status x *Wolbachia* status | 1 | 1 | 6.02 | 53.69 | <.0001* |
| Rep x DENV status x *Wolbachia* status | 5 | 5 | 0.43 | 0.77 | 0.57 |
