# Supplementary material for: Microbes increase thermal sensitivity in the mosquito Aedes aegypti, with the potential to change disease distributions
Source: PLoS Negl Trop Dis. 2021 Jul 22;15(7):e0009548. doi: 10.1371/journal.pntd.0009548 (PMC8297775; doi:10.1371/journal.pntd.0009548)
Supplement: S4 Table — ANOVAs for knockdown time for Fig 4 for each of the six individual temporal replicates with ‘DENV status’ and ‘Wolbachia status’ as factors. (DOCX) [file pntd.0009548.s004.docx]

**Supplemental Table 4. Impact of DENV and *Wolbachia* co-infection on KD time for each replicate independently.** ANOVAs for KD time for Fig. 4 for each of the six individual temporal replicates with ‘DENV status’ and ‘Wolbachia status’ as factors.

Rep 1: one-way ANOVA

| **Effect** | **Nparm** | ***df*** | **Sum of**  **Squares** | **F-Ratio** | ***p*-value** |
| --- | --- | --- | --- | --- | --- |
| Wolbachia status | 1 | 1 | 1752724 | 3.91 | 0.0555 |
| DENV status | 1 | 1 | 2805498 | 28.54 | <.0001* |

Rep 2: one-way ANOVA

| **Effect** | **Nparm** | ***df*** | **Sum of**  **Squares** | **F-Ratio** | ***p*-value** |
| --- | --- | --- | --- | --- | --- |
| Wolbachia status | 1 | 1 | 7782764 | 34.01 | <.0001* |
| DENV status | 1 | 1 | 12648125 | 55.28 | <.0001* |

Rep 3: one-way ANOVA

| **Effect** | **Nparm** | ***df*** | **Sum of**  **Squares** | **F-Ratio** | ***p*-value** |
| --- | --- | --- | --- | --- | --- |
| Wolbachia status | 1 | 1 | 1095610.0 | 5.25 | 0.027* |
| DENV status | 1 | 1 | 3469210.0 | 16.62 | 0.00020* |

Rep 4: one-way ANOVA

| **Effect** | **Nparm** | ***df*** | **Sum of**  **Squares** | **F-Ratio** | ***p*-value** |
| --- | --- | --- | --- | --- | --- |
| Wolbachia status | 1 | 1 | 7815277 | 31.44 | <.0001* |
| DENV status | 1 | 1 | 11853064 | 47.68 | <.0001* |

Rep 5: one-way ANOVA

| **Effect** | **Nparm** | ***df*** | **Sum of**  **Squares** | **F-Ratio** | ***p*-value** |
| --- | --- | --- | --- | --- | --- |
| Wolbachia status | 1 | 1 | 244314 | 10.15 | <0.0030* |
| DENV status | 1 | 1 | 12192576 | 50.65 | <0.00010* |

Rep 6: one-way ANOVA

| **Effect** | **Nparm** | ***df*** | **Sum of**  **Squares** | **F-Ratio** | ***p*-value** |
| --- | --- | --- | --- | --- | --- |
| Wolbachia status | 1 | 1 | 2718141.8 | 20.09 | <.0001* |
| DENV status | 1 | 1 | 7240768.6 | 53.52 | <.0001* |
