# Supplementary material for: Microbes increase thermal sensitivity in the mosquito Aedes aegypti, with the potential to change disease distributions
Source: PLoS Negl Trop Dis. 2021 Jul 22;15(7):e0009548. doi: 10.1371/journal.pntd.0009548 (PMC8297775; doi:10.1371/journal.pntd.0009548)
Supplement: S5 Table — Tukey’s post hoc comparisons of KD time for Fig 4 for all combinations of DENV (D+/-) and Wolbachia (W+/-) infection status. (DOCX) [file pntd.0009548.s005.docx]

**Supplemental Table 5. Impact of DENV and *Wolbachia* infections in mono and co-infection on KD time for each replicate independently**. Tukey’s post hoc comparisons of KD time for Fig. 4 for all combinations of DENV (D+/-) and *Wolbachia* (W+/-) infection status.

Adjusted *p*-value

|  | **Rep 1** | **Rep 2** | **Rep 3** | **Rep 4** | **Rep 5** | **Rep 6** |
| --- | --- | --- | --- | --- | --- | --- |
| D+*W*- vs. D-*W*- | <.0001* | <.0001* | <.0001* | <.0001* | <.0001* | <.0001* |
| D+*W*- vs. D+*W*+ | 0.26 | 0.71 | 0.43 | 0.99 | 0.90 | 0.29 |
| D+*W*- vs. D-*W*+ | 0.096 | 0.76 | 0.59 | 0.83 | 0.041* | 0.18 |
| D-*W*- vs. D+*W*+ | 0.0001* | <.0001* | 0.0004* | <.0001* | <.0001* | <.0001* |
| D-*W*- vs. D-*W*+ | 0.0006* | <.0001* | 0.0002* | <.0001* | <.0001* | <.0001* |
| D+*W*+ vs. D-*W*+ | 0.95 | 0.19 | 0.99 | 0.68 | 0.17 | 0.99 |
