# Supplementary material for: Microbes increase thermal sensitivity in the mosquito Aedes aegypti, with the potential to change disease distributions
Source: PLoS Negl Trop Dis. 2021 Jul 22;15(7):e0009548. doi: 10.1371/journal.pntd.0009548 (PMC8297775; doi:10.1371/journal.pntd.0009548)
Supplement: S6 Table — Summary of individual rep correlations between Wolbachia load and KD time for Fig 4. (DOCX) [file pntd.0009548.s006.docx]

**Supplemental Table 6. Correlation between *Wolbachia* load and KD time.** Summary of individual rep correlations between *Wolbachia* load and KD time for Fig. 4.

| **Rep** | ***n*** | **Pearson’s correlation (r)** | ***p*-value** |
| --- | --- | --- | --- |
| 1 | 18 | 0.052 | 0.84 |
| 2 | 20 | 0.23 | 0.32 |
| 3 | 20 | -0.24 | 0.305 |
| 4 | 18 | 0.16 | 0.51 |
| 5 | 20 | 0.32 | 0.16 |
| 6 | 20 | -0.33 | 0.15 |
