# Supplementary material for: Microbes increase thermal sensitivity in the mosquito Aedes aegypti, with the potential to change disease distributions
Source: PLoS Negl Trop Dis. 2021 Jul 22;15(7):e0009548. doi: 10.1371/journal.pntd.0009548 (PMC8297775; doi:10.1371/journal.pntd.0009548)
Supplement: S7 Table — Summary of t-tests from pilot experiments conducted at varying temperatures showing average time to knockdown for dengue infected (D+W-) and uninfected (D-W-) individuals. All temperatures resulted in a knockdown phenotype, but 42°C led to death in 97% of individuals trialed in the WT line even without the presence of DENV. 42°C also allowed resulted in faster knockdown times during the assay. (DOCX) [file pntd.0009548.s007.docx]

**Supplemental Table 7. Survival during pilot knockdowns at varying temperatures (DENV+/-).** Summary of t-tests from pilot experiments conducted at varying temperatures showing average time to knockdown for dengue infected (D+*W*-) and uninfected (D-*W*-) individuals. All temperatures resulted in a knockdown phenotype, but 42°C led to death in 97% of individuals trialed in the WT line even without the presence of DENV. 42°C also allowed resulted in faster knockdown times during the assay.

| **Knockdown (KD) Temperature** | **Percent survival after knockdown** | **D+*W*-  Average**  **KD time (sec)** | **D-*W*-**  **Average**  **KD time (sec)** | ***df*** | **F-Ratio** | ***p*-value** |
| --- | --- | --- | --- | --- | --- | --- |
| 39°C | 10% | 1021 | 2414 | 35 | 4.66 | 0.0041 |
| 40°C | 11% | 581.6 | 2422 | 6 | 15.32 | 0.0065 |
| 42°C | 3% | 168.7 | 1170 | 11 | 343.20 | 0.0254 |
