# Supplementary material for: Microbes increase thermal sensitivity in the mosquito Aedes aegypti, with the potential to change disease distributions
Source: PLoS Negl Trop Dis. 2021 Jul 22;15(7):e0009548. doi: 10.1371/journal.pntd.0009548 (PMC8297775; doi:10.1371/journal.pntd.0009548)
Supplement: S8 Table — (DOCX) [file pntd.0009548.s008.docx]

**Supplemental Table 8. Primers and probes for dengue virus serotype 2 (strain ET300, Genbank EF440433**)

| **Target Genomic Region** | **Direction** | **5’-3’ sequence** | **Primer Melting Point** |
| --- | --- | --- | --- |
| 3’ UTR – **POLY gene** | Forward | AAGGACTAGAGGTTAGAGGAGACCC | 54ºC |
|  | Reverse | CGTTCTGTGCCTGGAATGATG | 58ºC |
|  | Probe: FAM | AACAGCATATTGACGCTGGGAGAGACCAGA-BHQ1 |  |
