# Supplementary material for: Microbes increase thermal sensitivity in the mosquito Aedes aegypti, with the potential to change disease distributions
Source: PLoS Negl Trop Dis. 2021 Jul 22;15(7):e0009548. doi: 10.1371/journal.pntd.0009548 (PMC8297775; doi:10.1371/journal.pntd.0009548)
Supplement: S1 Fig — KD time (seconds) vs. DENV load (per mosquito) in Wildtype (W-) mosquitoes. Each point represents a single mosquito. Data include all individuals across 6 replicate experiments. There was no significant relationship (Pearson’s correlation, P = 0.204) between KD time and DENV load. (DOCX) [file pntd.0009548.s009.docx]

**Supplemental Figure 1. Relationship between viral load and thermal knockdown in mosquitoes for Fig. 4.**
