# Supplementary material for: Microbes increase thermal sensitivity in the mosquito Aedes aegypti, with the potential to change disease distributions
Source: PLoS Negl Trop Dis. 2021 Jul 22;15(7):e0009548. doi: 10.1371/journal.pntd.0009548 (PMC8297775; doi:10.1371/journal.pntd.0009548)
Supplement: S2 Fig — Average Wolbachia loads with (D+W+) and without (D-W+) DENV. Data are pooled across 6 replicate experiments. Graphs depict mean ± sem Wolbachia per host cell. Wolbachia load is reduced in the presence of DENV infection (df = 116, F-Ratio = 1.92, p<0.0001). (DOCX) [file pntd.0009548.s010.docx]

**Supplemental Figure 2. Reduction of *Wolbachia* load in the presence of DENV for Fig. 4.**

*******
