# Supplementary material for: Microbes increase thermal sensitivity in the mosquito Aedes aegypti, with the potential to change disease distributions
Source: PLoS Negl Trop Dis. 2021 Jul 22;15(7):e0009548. doi: 10.1371/journal.pntd.0009548 (PMC8297775; doi:10.1371/journal.pntd.0009548)
Supplement: S3 Fig — Average DENV loads for Wolbachia infected (D+W+) and uninfected (D+W-). Data are pooled across the 6 replicate experiments. Graphs depict mean ± standard error. Loads are reduced in the presence of Wolbachia (df = 115, F = 13.32, p<0.001). (DOCX) [file pntd.0009548.s011.docx]

**Supplemental Figure 3. Evidence of *Wolbachia*-mediated blocking of DENV for Fig. 4**

*******
